# Machine learning model for predicting the cold–heat pattern in Kampo medicine: a multicenter prospective observational study

**DOI:** 10.3389/fphar.2024.1412593

**Published:** 2024-10-25

**Authors:** Ayako Maeda-Minami, Tetsuhiro Yoshino, Kotoe Katayama, Yuko Horiba, Hiroaki Hikiami, Yutaka Shimada, Takao Namiki, Eiichi Tahara, Kiyoshi Minamizawa, Shin-Ichi Muramatsu, Rui Yamaguchi, Seiya Imoto, Satoru Miyano, Hideki Mima, Kazushi Uneda, Tatsuya Nogami, Koichi Fukunaga, Kenji Watanabe

**Affiliations:** ^1^ Faculty of Pharmaceutical Sciences, Tokyo University of Science, Chiba, Japan; ^2^ Center for Kampo Medicine, Keio University School of Medicine, Shinjuku, Tokyo, Japan; ^3^ Human Genome Center, the Institute of Medical Science, University of Tokyo, Minato, Tokyo, Japan; ^4^ Shikino Care Center, Toyama, Japan; ^5^ University of Toyama, Toyama, Japan; ^6^ Department of Japanese Oriental (Kampo) Medicine, Graduate School of Medicine, Chiba University, Chiba, Japan; ^7^ Department of Kampo Medicine, Aizu Medical Center, Fukushima Medical University, Fukushima, Japan; ^8^ Department of Oriental Medicine, Kameda Medical Center, Chiba, Japan; ^9^ Division of Oriental Medicine, Center of Community Medicine, Jichi Medical University, Tochigi, Japan; ^10^ Division of Cancer Systems Biology, Aichi Cancer Center Research Institute, Nagoya, Aichi, Japan; ^11^ Department of Integrated Analytics, M&D Data Science Center, Tokyo Medical and Dental University, Bunkyo, Tokyo, Japan; ^12^ Promoting Organization for Future Creators, Kyushu University, Fukuoka, Japan; ^13^ Department of Kampo Medicine, Tokai University School of Medicine, Isehara, Kanagawa, Japan; ^14^ Department of Medicine, Division of Pulmonary Medicine, Keio University School of Medicine, Shinjuku, Tokyo, Japan

**Keywords:** Kampo medicine, the International Classification of Diseases, tangled heat/cold pattern, moderate (heat/cold) pattern, prediction model

## Abstract

**Objective:**

The purpose of this study was to predict the four cold–heat patterns in patients who have the subjective symptoms of the cold–heat pattern described in the International Classification of Diseases Traditional Medicine Conditions – Module 1 by applying a machine learning algorithm.

**Methods:**

Subjects were first-visit Kampo outpatients at six institutions who agreed to participate in this multicenter prospective observational study. The cold pattern model and the heat pattern model were created separately with 148 symptoms, body mass index, blood pressure (systolic and diastolic), age, and sex. Along with a single cold or heat pattern, the tangled heat/cold pattern is defined as being predicted by both cold and heat patterns, while the moderate (heat/cold) pattern is defined as being predicted by neither the cold pattern nor the heat pattern.

**Results:**

We included 622 participants (mean age ±standard deviation, 54.4 ± 16.9; with female 501). The accuracy, macro-recall, precision, and F1-score of a combination of the two prediction models were 96.7%, 93.2%, 85.6%, and 88.5% respectively. The important items were compatible with the definitions of the cold–heat pattern.

**Conclusion:**

We developed a prediction model on cold–heat patterns with data from patients whose subjective cold/heat-related symptoms matched the cold–heat pattern diagnosis by the physician.

## 1 Introduction

The 11th revision of the International Classification of Diseases (ICD-11), effective January 2022 (World Health Organization, 2023), includes for the first time a chapter on traditional medicine conditions— Module 1 (ICD-11TM1)—including traditional East Asian medicine practiced mainly in China, Korea, and Japan. Kampo diagnosis is based on a synthesis of findings obtained through inspection, listening and smell, inquiring, and palpation, and relies heavily on the physician’s five senses, experience, and tacit knowledge. The selection of an appropriate Kampo medicine is based on traditional Kampo diagnosis. Even for a single conventional biomedicine diagnosis, such as perimenopausal symptoms or atopic dermatitis, Kampo specialists use various treatment options based on pattern diagnosis such as a heat–cold pattern. With the inclusion of Kampo diagnosis in ICD-11, it is necessary to visualize and quantify subjective information on the Kampo specialist involved in the diagnosis, which has rarely been done, as reproducible information.

In Japan, a single medical license allows physicians to prescribe both conventional biomedicines and Kampo medicines, and over 90% of physicians have experience prescribing Kampo medicines (Moschik et al., 2012). However, most physicians are not specialists in Kampo medicine and lack detailed knowledge of it. Therefore, it is crucial for non-specialists in Kampo medicine to easily grasp the patient’s traditional pattern diagnosis when choosing an appropriate Kampo medicine.

We have developed a model to predict a deficiency–excess and cold–heat pattern from patient questionnaires and have identified important items to predict the deficiency–excess and cold–heat pattern (Katayama et al., 2014; Maeda-Minami et al., 2019; Maeda-Minami et al., 2020). We have also identified the important items for the predicting deficiency–excess and cold–heat pattern (Katayama et al., 2014; Maeda-Minami et al., 2019; Maeda-Minami et al., 2020). In addition to our previous study, there have been previous reports on prediction models for the cold–heat pattern (Lee B. J. et al., 2018; Lee J. et al., 2018).

In Japan, there is a moderate (heat/cold) pattern in addition to the cold pattern, heat pattern, and tangled heat/cold pattern in the cold–heat pattern (Yakubo et al., 2014; Maeda-Minami et al., 2021). A moderate (heat/cold) pattern is defined in ICD-11TM1 as “a pattern characterized by the absence of findings that indicate the heat pattern (TM), such as heat intolerance, red complexion, and hot limbs, or cold pattern (TM), such as cold intolerance, pale complexion, and cold limbs. It may be explained by the average level of metabolic activity” (World Health Organization, 2023). However, the cold–heat pattern prediction models in previous studies have been able to predict only the cold and heat patterns among the four cold–heat patterns (Katayama et al., 2014; Maeda-Minami et al., 2020; Lee BJ et al., 2018; Lee J et al., 2018). No model predicts the tangled heat/cold pattern and moderate (heat/cold) pattern as well.

In addition, in constructing the cold–heat pattern prediction model, we investigated our data carefully and found that the ICD-11TM1 definition of the cold–heat pattern includes many complaints about patients’ cold–heat pattern, but the patients’ cold–heat pattern complaints did not match the physician’s diagnosis of the cold–heat pattern. The physician’s diagnosis of the cold–heat pattern and the patient’s symptoms of the cold–heat pattern were consistent in only 33.6% of all patients. We have also reported that using training data with smaller deviations from the definition leads to models that are more realistic and have a higher accuracy rather than using pragmatic data that include a wider variety of patients (Maeda-Minami et al., 2022).

The purpose of this study was to predict the four cold–heat patterns in patients who have the subjective symptoms of the cold–heat pattern described in ICD-11TM1 by applying a machine learning algorithm.

## 2 Methods

This is a multicenter prospective observational study.

### 2.1 Participants

Subjects were first-visit Kampo outpatients at six institutions, Chiba University, Iizuka Hospital, Keio University, University of Toyama, Kameda General Hospital, and Jichi Medical University, who agreed to participate in the study. The data collection periods for each institution were as follows: Chiba University from March 2012 to January 2015, Iizuka Hospital from January 2012 to February 2015, Keio University from June 2012 to March 2013, University of Toyama from June 2012 to February 2015, Kameda General Hospital from January 2012 to March 2013, and Jichi Medical University was from January 2012 to March 2012. From previous studies, we found no remarkable differences among the six institutions in terms of participants’ demographic characteristics, including mean age, female-to-male ratio, mean body mass index (BMI), mean blood pressure, physician-diagnosed patterns, and important items in the prediction models in previous studies using the same dataset. Therefore, data from all institutions were combined for this present analysis (Maeda-Minami et al., 2019; Maeda-Minami et al., 2020).

Exclusion criteria were various data deficits (blood pressure, height, weight, etc.), age less than 20 years, fewer than 20 questionnaire items answered, and discrepancy in the cold–heat pattern between the physician’s diagnosis and the patient’s symptom. We have already reported that it was difficult to predict the pattern from the questionnaire items alone in patients with fewer than 20 items (Katayama et al., 2014; Maeda-Minami et al., 2019; Maeda-Minami et al., 2020).

The patients were selected based on their responses to the cold–heat pattern questionnaire, referring to the definition in ICD-11TM1 (World Health Organization, 2023). A part of the questionnaire was administered to patients using the visual analog scale (VAS, 0–100 mm). A VAS value of 0 was considered to indicate that the patient responded that he/she did not have the symptom, and a VAS value greater than 0 was considered to indicate that the patient responded that he/she had the symptom. The cold–heat pattern was determined from the patient’s symptoms, according to the classification in Table 1.

**TABLE 1:** Pattern determined from the patient’s symptom.

Pattern of judgment	Cold pattern	Moderate (heat/cold) pattern	Heat pattern	Tangled heat/cold pattern
VAS of cold pattern’s items	At least one item’s VAS was greater than 0	All items’ VAS were 0	All items’ VAS were 0	At least one item’s VAS was greater than 0
VAS of heat pattern’s items	All items’ VAS were 0	All items’ VAS were 0	At least one item’s VAS was greater than 0	At least one item’s VAS was greater than 0

VAS, visual analog scale.

Only those patients were selected for whom the pattern determined from the patient’s symptoms regarding ICD-11TM1 matched the pattern diagnosed by the physician. We used the traditional pattern diagnosis diagnosed by specialists in Kampo medicine as the ground truth. The specialists in Kampo medicine employed in this study had held active biomedical licenses, completed residencies such as internal medicine or obstetrics and gynecology, and were board-certified by the Japan Society for Oriental Medicine with 3 or more years of clinical fellowship training. We ensured consistency by also restricting patients whose responses to interview items met the ICD-11TM1 definition.

### 2.2 Patient medical information items

A total of 154 medical information items were obtained from patients, 148 interview items (including 29 binary items and 119 VAS items), vital data [height, weight, and blood pressure (systolic and diastolic)], and attribute data (age and sex). Since BMI was the most important item in the prediction model for the deficiency–excess pattern in previous studies (Katayama et al., 2014; Maeda-Minami et al., 2019; Maeda-Minami et al., 2020), height(m) and weight (kg) were converted to BMI instead of using them as they were when creating the prediction model. Therefore, the independent variables used in the prediction models were 153 items.

### 2.3 Construction of a cold–heat pattern prediction model using machine learning

#### 2.3.1 Training algorithm

For the construction of the cold pattern prediction model and the heat pattern prediction model, the algorithm used a random forest classification derived by Breiman (1996), Breiman (2001), and Breiman et al. (2008). Random forest generates multiple sample datasets from the training data using the bootstrap method and constructs decision trees for each dataset (Breiman, 2001). Each decision tree is independently trained, using a random subset of features to determine the branching points (Breiman, 2001). This ensemble learning method combines multiple decision trees for prediction (Breiman, 2001). The classification algorithm used in this study makes predictions on unknown data by taking the majority vote of each decision tree’s classification result (Breiman, 2001). Results are expressed as vote values ranging from 0 to 1. We have previously reported that random forests had the best accuracy for predicting deficiency–excess and cold–heat patterns (Katayama et al., 2014; Maeda-Minami et al., 2019; Maeda-Minami et al., 2020). The parameters for random forest include the number of decision trees, the number of features used for each tree, and the number of samples in the end nodes, which were set to 500, 12, and 1, respectively.

By creating a random forest model, we can determine the importance of each item in the prediction, which is calculated based on the decrease in the Gini coefficient when the variable is excluded from the model (mean decrease Gini). Higher values indicate that the item significantly contributes to the prediction.

#### 2.3.2 Explanatory variables

The explanatory variables for the prediction models were 148 symptoms (including 29 binary items and 119 VAS items), BMI, blood pressure (systolic and diastolic), age, and sex, for a total of 153 items. VAS values were normalized by dividing each VAS value by the maximum VAS value for each patient, as in previous studies (Katayama et al., 2011; Katayama et al., 2014; Maeda-Minami et al., 2019; Maeda-Minami et al., 2020).

#### 2.3.3 Training data sampling

We have already reported the confounding relationship between the deficiency–excess and cold–heat patterns (Maeda-Minami et al., 2020). In our data, there were approximately nine times as many patients with deficiency and cold patterns as patients with deficiency and heat patterns and approximately twice as many patients with excess and heat patterns as patients with excess and cold patterns. Our data had both high-frequency and low-frequency classes, which influenced this relationship. When creating prediction models using imbalanced data, it is generally suggested to sample the training data according to either the high-frequency or low-frequency class (Fujiwara et al., 2020; He and Garcia, 2009). Our previous study found that random undersampling to match the low-frequency class, rather than extracting based on the overall data ratio, led to better prediction models with key items closely aligning with the ICD-11TM1 definition (Maeda-Minami et al., 2020).

Following the method of previous research, the training data were randomly sampled from the following 12 pattern patients with a balance of 10 cases each; deficiency-cold pattern, deficiency-moderate (heat/cold) pattern, deficiency-heat pattern, deficiency-tangled heat/cold pattern, medium-cold pattern, medium-moderate (heat/cold) pattern, medium-heat pattern, medium-tangled heat/cold pattern, excess-cold pattern, excess-moderate (heat/cold) pattern, excess-heat pattern, and excess-tangled heat/cold pattern (Table 2). The number of patients in the excess-moderate (heat/cold) pattern was less than 10, so bootstrap resampling was performed. We sampled a total of 120 cases from 12 patterns and divided them into 60 cases each to train classification models for the cold pattern or not and heat pattern or not. Based on our previous results, this was considered sufficient for training data. The remaining data other than the training data were used as test data.

**TABLE 2:** Training and test data.

Total data	Cold pattern	Moderate (heat/cold) pattern	Heat pattern	Tangled heat/cold pattern	Total
Deficiency pattern	169	17	16	82	284
Medium pattern	82	27	30	95	234
Excess pattern	16	4	34	50	104
Total	267	48	80	227	622
Training data					
Deficiency pattern	10	10	10	10	40
Medium pattern	10	10	10	10	40
Excess pattern	10	10	10	10	40
Total	30	30	30	30	120
Test data					
Deficiency pattern	159	7	6	72	244
Medium pattern	72	17	20	85	194
Excess pattern	6	0	24	40	70
Total	237	24	50	197	508

#### 2.3.4 Combining two models to predict the cold–heat pattern

**FIGURE 1:**
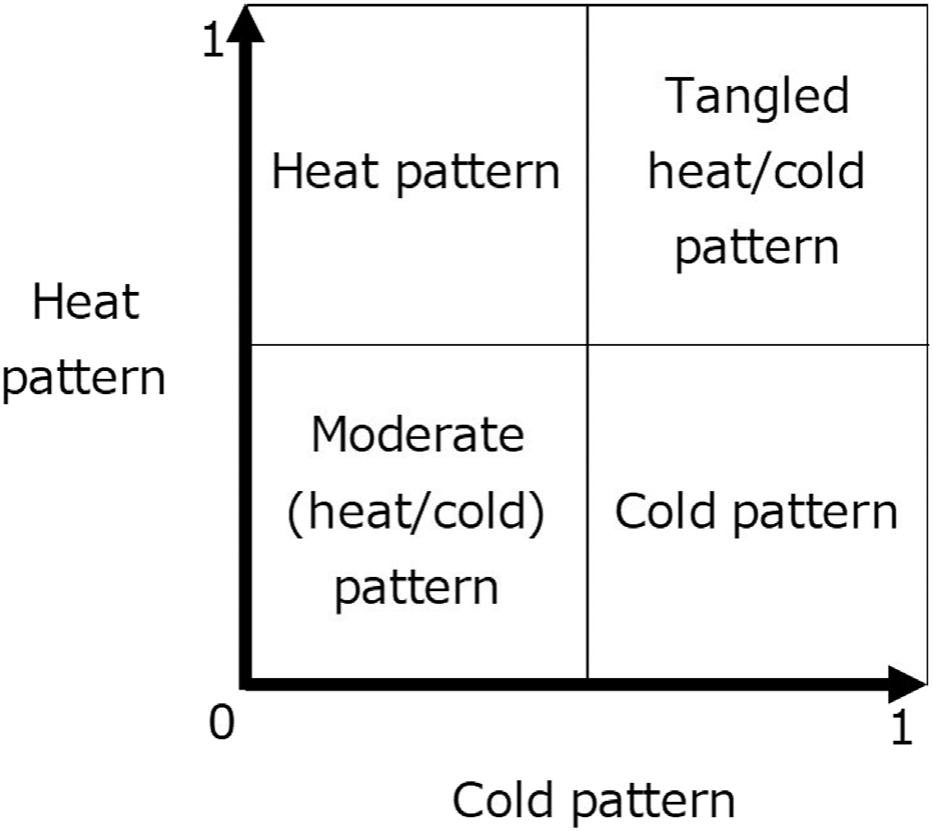
Relationship between the pattern and vote of the cold–heat pattern prediction model.

The cold pattern and heat pattern models were created separately for this study to predict the four cold–heat patterns (Figure 1). In ICD-11, the tangled heat/cold pattern is described as having both cold and heat patterns, while the moderate (heat/cold) pattern is described as having neither a cold pattern nor a heat pattern (World Health Organization, 2023). The cold pattern prediction model and the heat pattern prediction model were constructed by the random forest classification algorithm using the training data.

#### 2.3.5 Objective variables

At the stage of creating the cold pattern prediction model, data diagnosed by Kampo specialists as the cold pattern or tangled heat/cold pattern were used as 1/true, while data diagnosed as the heat pattern or moderate (heat/cold) pattern were used as 0/false for training. Similarly, data diagnosed by Kampo specialists as the heat pattern or tangled heat/cold pattern were used as 1/true, while data diagnosed as the cold pattern or moderate (heat/cold) pattern were used as 0/false for the heat pattern prediction model.

#### 2.3.6 Evaluating prediction performance

Both prediction models are used to calculate a vote value in the range of 0–1 for each test data (Figure 1). In the cold pattern prediction model, the calculated vote value of 0.5 or more was judged as a cold pattern or tangled heat/cold pattern and that of less than 0.5 as a heat pattern or moderate (heat/cold) pattern. In the heat pattern prediction model, a calculated vote value of 0.5 or higher was considered a heat pattern or tangled heat/cold pattern, and a calculated vote value of less than 0.5 was considered a cold pattern or moderate (heat/cold) pattern.

Based on the results of the cold pattern prediction model and the heat pattern prediction model, we determined the four cold–heat patterns as the cold pattern, moderate (heat/cold) pattern, heat pattern, and tangled heat/cold pattern for each of the test data. We calculated the accuracy (percent agreement) between the ground truth and the patterns predicted by the prediction models. We also calculated specific metrics used in multi-class classification, macro-recall, precision, and F1-score.

The 30 most important items for the cold pattern prediction model and the heat pattern prediction model were selected. Moreover, before finalizing the random forest, we repeated random samplings to create the models multiple times and confirmed that it generally resulted in similar accuracy and important explanatory items each time.

### 2.4 Software

All analyses were performed using R4.2.2 (R Foundation for Statistical Computing, Vienna, Austria). R’s random forests package was used (Breiman et al., 2008).

## 3 Results

### 3.1 Participants

**FIGURE 2:**
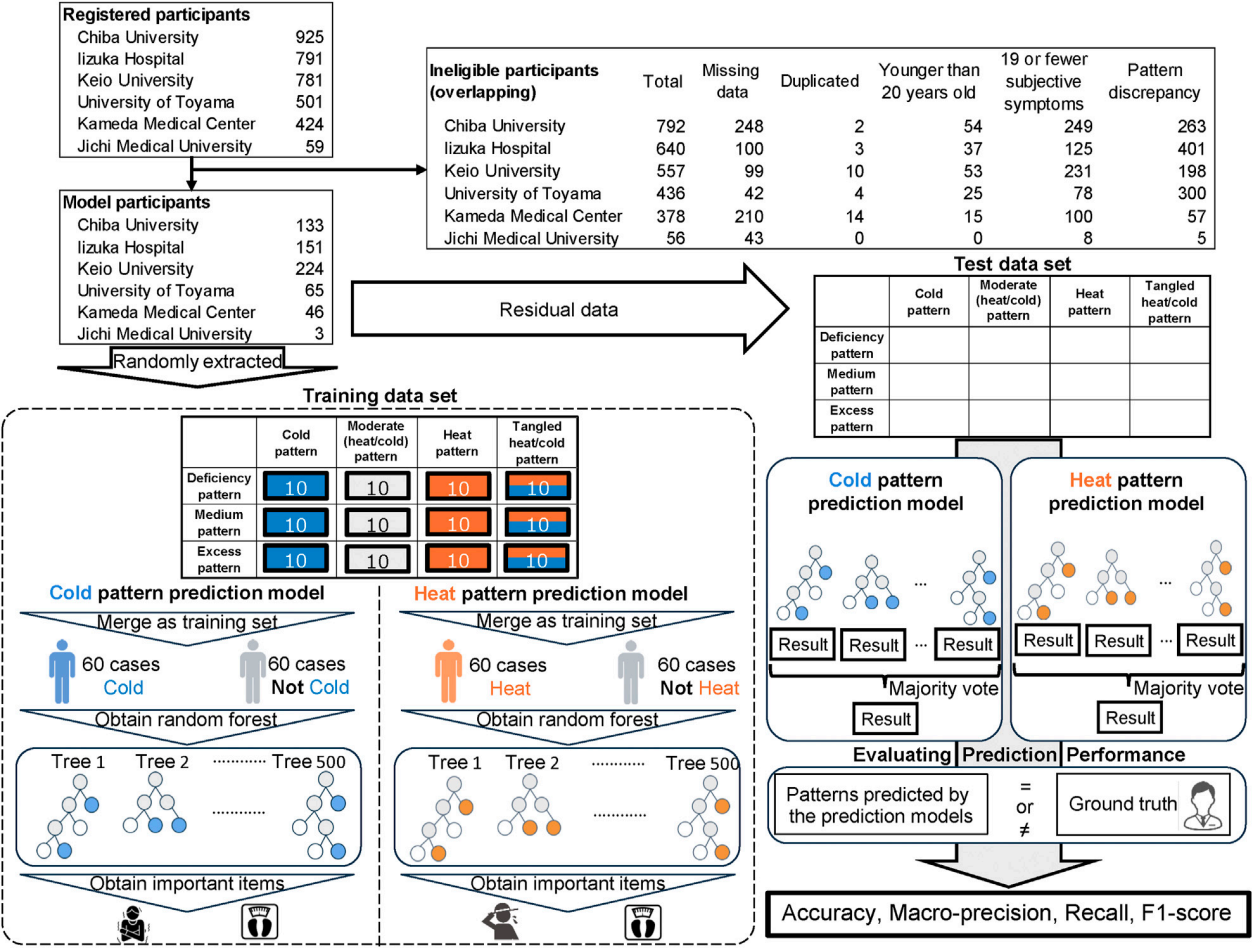
Flow chart for inclusion/exclusion and model construction/evaluation.

The number of patients registered at the six institutions was 925 at Chiba University, 791 at Iizuka Hospital, 781 at Keio University, 501 at University of Toyama, 424 at Kameda General Hospital, and 59 at Jichi Medical University (Figure 2). The number of eligible patients was 133 at Chiba University, 151 at Iizuka Hospital, 224 at Keio University, 65 at University of Toyama, 46 at Kameda General Hospital, and 3 at Jichi Medical University. The patient backgrounds used in the prediction model are shown in Table 3. The cross table on the deficiency–excess pattern and cold–heat pattern diagnosed by the physicians is shown in Table 2.

**TABLE 3:** Background of model participants.

Characteristics	numbers
Age (year) (mean ± SD)	54.4 ± 16.9
Male: female	121 : 501
BMI, kg/m^2^ (Mean ± SD)	21.7 ± 3.7

BMI, body mass index; SD, standard deviation

### 3.2 Cold–heat pattern prediction including the tangled heat/cold and moderate (heat/cold) patterns

The accuracy of the cold pattern prediction model was 96.7%, and the accuracy of the heat pattern prediction model was 96.7%. The accuracy, macro-recall, precision, and F1-score by the combination of the two prediction models were 96.7%, 93.2%, 85.6%, and 88.5%, respectively (Table 4).

**TABLE 4:** Prediction results for four cold–heat patterns.

		Physician’s diagnosis				
		Cold pattern	Moderate (heat/cold) pattern	Heat pattern	Tangled heat/cold pattern	Total
Prediction	Cold pattern	230	0	0	10	240
	Moderate (heat/cold) pattern	6	24	6	0	36
	Heat pattern	0	0	43	10	53
	Tangled heat/cold pattern	1	0	1	177	179
	Total	237	24	50	197	508

The important items in the cold pattern prediction model include cold hypersensitivity in legs, cold intolerance, cold hypersensitivity in hands, general cold hypersensitivity, and blood pressure. The important items in the heat pattern prediction model include heat hypersensitivity in face, easy to sweat, heat intolerance, night sweats, and hot flashes. These items are included in the definitions of the cold–heat pattern in the ICD-11TM1 (Table 5).

**TABLE 5:** Important items for the cold pattern prediction model and heat pattern prediction model.

Order	Cold pattern		Heat pattern	
	Item	Importance	Item	Importance
1	Cold hypersensitivity in legs	8.86	Heat hypersensitivity in face	7.32
2	Cold intolerance	8.48	Easy to sweat	6.54
3	Cold hypersensitivity in hands	2.92	Heat intolerance	6.21
4	General cold hypersensitivity	1.66	Night sweats	2.55
5	Diastolic blood pressure	1.11	Hot flashes	2.14
6	Neck stiffness	0.98	Age	1.19
7	Systolic blood pressure	0.88	Depressed mood	1.12
8	BMI	0.86	Systolic blood pressure	0.93
9	Difficulty falling asleep	0.78	Menstrual pain	0.88
10	Chest pain	0.78	Blot	0.87
11	Stomach fullness	0.78	Diastolic blood pressure	0.81
12	Back stiffness	0.73	Dry skin	0.70
13	Eyestrain	0.73	Feeling sluggish	0.63
14	Shoulder stiffness	0.73	Knee pain	0.61
15	Age	0.71	Irritated	0.61
16	Thirsty	0.64	BMI	0.60
17	Easily fatigued	0.64	Facial edema	0.60
18	Drink water often	0.62	Bleary eyes	0.59
19	Flatulence	0.58	Palpitations	0.57
20	Hand numbness	0.58	Neck stiffness	0.52
21	Depressed mood	0.58	Lower back stiffness	0.51
22	Arousal during sleep	0.54	Headache	0.50
23	Cold hypersensitivity in lower back	0.54	Easily fatigued	0.48
24	Throat pain	0.51	Itchy skin	0.48
25	Legs spasms	0.50	Heat hypersensitivity in hands	0.48
26	Short attention span	0.47	Shoulder stiffness	0.45
27	Dry mouth	0.46	Drink water often	0.44
28	Burping	0.44	Sneezing	0.44
29	Heat hypersensitivity in face	0.43	Stuffy nose	0.44
30	Light headedness	0.42	Forgetfulness	0.42

BMI, body mass index.

## 4 Discussions

We developed prediction models on four cold–heat patterns including the tangled heat/cold and moderate (heat/cold) patterns at six institutions specializing in Kampo in Japan. First, we constructed a cold pattern prediction model and a heat pattern prediction model and then combined them to predict the four cold–heat patterns. Even though the model was validated by the selected patients’ data, the accuracy was over 90%, showing high concordance with the physician’s diagnosis. The high accuracy suggests that the combination of two prediction models is likely to be a successful method for predicting the cold–heat pattern.

In the present study, we combined the two prediction models to predict a physician’s cold–heat pattern diagnosis, meaning both models have to predict the diagnosis correctly to reach a correct conclusion. There was no case of both models predicting the wrong diagnosis (i.e., predicted as a moderate (heat/cold) pattern for cases with a tangled heat/cold pattern by specialist physicians). Most of the misclassified cases occurred because one of the prediction models failed to identify a cold or heat pattern in patients diagnosed by physicians as having such patterns. In only two cases, one of the two prediction models incorrectly identified a cold or heat pattern in patients who were diagnosed by physicians as having no such pattern. In both cases, our model concluded a tangled heat/cold pattern, while the specialists diagnosed them as cold or heat.

The prediction model was constructed based on the results of the patient questionnaires, but we also found that the accuracy of the prediction model for the deficiency–excess pattern increased when the results of abdominal strength diagnosed by the physician were added as an explanatory variable (Maeda-Minami et al., 2019; Maeda-Minami et al., 2020). By adding the results of the physician’s examination, for example, complexion and limb temperature, according to the ICD-11TM1 definition of cold/heat patterns, as an explanatory variable may enable the construction of a model with high accuracy even when using patients whose patterns did not match the patterns diagnosed by the physician based on the patient’s symptoms. However, since one of the purposes of our study is to create a diagnostic aid tool that will allow physicians who do not specialize in Kampo to easily make an appropriate diagnosis and select a Kampo medicine, we did not incorporate the results of the physician’s examination into our study.

This study has several limitations. The prediction models were constructed excluding patients whose patterns diagnosed by the physician did not agree with subjective symptoms, and the accuracy was approximately 10% to these patients (data not shown). Further research is needed to use predictive models in daily clinical practice. To improve the predictive model that can also be applied to the patients excluded in this study, we plan to apply the current model in real clinical settings, examine which types of patients it misclassifies, and incorporate explanatory variables that can be measured even by non-specialists in Kampo medicine. These results cannot be applied to those under 20 years of age because those under 20 years of age were excluded from this study. Similarly, patients with 19 or fewer symptoms are excluded from the present study due to the difficulty of predicting their pattern diagnosis with our data according to our prior studies for the deficiency–excess pattern. In our previous study, the most important items that contributed to the prediction of the cold–heat pattern in the prediction model was patients’ symptoms, so it was difficult to predict it accurately for patients with few symptoms. Since this study was limited to institutions in Japan that specialize in Kampo, it is unclear whether the results can be applied to other traditional East Asian medicine. Additionally, external validation was not conducted in this study even though we used the data from multiple facilities in various regions of Japan for model construction.

## 5 Conclusion

We developed a prediction model on cold–heat patterns with high accuracy, macro-recall, precision, and F1-score with data from patients whose subjective cold/heat-related symptoms matched the cold–heat pattern diagnosis by the physician.

## Data Availability

The data that support the findings of this study are not publicly available due to their containing information that could compromise the privacy of research participants but are available from the corresponding author TY upon reasonable request.
