# Correction: Machine learning model for predicting the cold–heat pattern in Kampo medicine: a multicenter prospective observational study

**DOI:** 10.3389/fphar.2026.1789439

**Published:** 2026-01-29

**Authors:** Ayako Maeda-Minami, Tetsuhiro Yoshino, Kotoe Katayama, Yuko Horiba, Hiroaki Hikiami, Yutaka Shimada, Takao Namiki, Eiichi Tahara, Kiyoshi Minamizawa, Shin-Ichi Muramatsu, Rui Yamaguchi, Seiya Imoto, Satoru Miyano, Hideki Mima, Kazushi Uneda, Tatsuya Nogami, Koichi Fukunaga, Kenji Watanabe

**Affiliations:** 1 Faculty of Pharmaceutical Sciences, Tokyo University of Science, Chiba, Japan; 2 Center for Kampo Medicine, Keio University School of Medicine, Shinjuku, Tokyo, Japan; 3 Human Genome Center, the Institute of Medical Science, University of Tokyo, Minato, Tokyo, Japan; 4 Shikino Care Center, Toyama, Japan; 5 University of Toyama, Toyama, Japan; 6 Department of Japanese Oriental (Kampo) Medicine, Graduate School of Medicine, Chiba University, Chiba, Japan; 7 Department of Kampo Medicine, Aizu Medical Center, Fukushima Medical University, Fukushima, Japan; 8 Department of Oriental Medicine, Kameda Medical Center, Chiba, Japan; 9 Division of Oriental Medicine, Center of Community Medicine, Jichi Medical University, Tochigi, Japan; 10 Division of Cancer Systems Biology, Aichi Cancer Center Research Institute, Nagoya, Aichi, Japan; 11 Department of Integrated Analytics, M&D Data Science Center, Tokyo Medical and Dental University, Bunkyo, Tokyo, Japan; 12 Promoting Organization for Future Creators, Kyushu University, Fukuoka, Japan; 13 Department of Kampo Medicine, Tokai University School of Medicine, Isehara, Kanagawa, Japan; 14 Department of Medicine, Division of Pulmonary Medicine, Keio University School of Medicine, Shinjuku, Tokyo, Japan

**Keywords:** Kampo medicine, the International Classification of Diseases, tangled heat/cold pattern, moderate (heat/cold) pattern, prediction model

In the published article, Affiliation 2 was erroneously omitted for author Ayako Maeda-Minami. This affiliation has now been added for author Ayako Maeda-Minami.

There was an error in the **Funding** statement. It was mistakenly declared that ‘An article processing charge and English editing fee for this article were paid by the Japan Agency for Medical Research and Development (JP23lk0310096 and JP24lk0310096).’ Instead, the authors confirm that an article processing charge was paid by a collaborative research program fund of Keio University and TSUMURA and CO.

The correct Funding statement appears below.

The author(s) declared that financial support was received for this work and/or its publication. This work was supported by a Grant-in-Aid for Research on Propulsion Study of Clinical Research from the Ministry of Health, Labor and Welfare in building the questionnaire and data collection. An article processing charge for this article was paid by a collaborative research program fund of Keio University and TSUMURA & CO. The funder was not involved in the study design, collection, analysis, interpretation of data, the writing of this article, or the decision to submit it for publication.

The **Conflict of interest** statement was erroneously given as ‘The authors declare that the research was conducted in the absence of any commercial or financial relationships that could be construed as a potential conflict of interest. The author(s) declared that they were an editorial board member of Frontiers, at the time of submission. This had no impact on the peer review process and the final decision.’

The correct Conflict of interest statement appears below.

Authors TY and AMM are employed at Keio University for collaborative research with TSUMURA & CO. Authors YH and TN (16th author) received a lecture fee from TSUMURA & CO., and Kracie, Ltd. Authors TN (seventh author) and KF received research grant support from TSUMURA & CO. Authors ET, SHM, and KW received a lecture fee from TSUMURA & CO. ET and KU received research grant support from TSUMURA & CO. and SEIRIN Co., Ltd.

The remaining authors declare that the research was conducted in the absence of any commercial or financial relationships that could be construed as a potential conflict of interest.

The author(s) declared that they were an editorial board member of Frontiers, at the time of submission. This had no impact on the peer review process and the final decision.

The original article has been updated.

