# Supplementary material for: Machine learning model for predicting the cold–heat pattern in Kampo medicine: a multicenter prospective observational study
Source: Front Pharmacol. 2024 Oct 25;15:1412593. doi: 10.3389/fphar.2024.1412593 (PMC11543495; doi:10.3389/fphar.2024.1412593)
Supplement: Supplementary file 1 [file Table1.DOCX]

Supplementary Table 1 Patient questionnaire items

| No. | Binary questions | No. | Questions with | No. | Questions with |
| --- | --- | --- | --- | --- | --- |
|  |  |  | visual analogue scales |  | visual analogue scales |
| 1 | Appetite loss / Good appetite | 1 | Difficulty falling asleep | 61 | General cold hypersensitivity |
| 2 | Slow / Fast speed of the meal | 2 | Arousal during sleep | 62 | Cold hypersensitivity in hands |
| 3 | I dream frequently | 3 | Early-morning awakening | 63 | Cold hypersensitivity in legs |
| 4 | Single volume of urine large / low | 4 | Daytime sleepiness | 64 | Cold hypersensitivity in lower back |
| 5 | Hard stool | 5 | Difficulty urinating | 65 | Heat hypersensitivity in face |
| 6 | Small and round stool | 6 | Urination pain | 66 | Heat hypersensitivity in hands |
| 7 | Soft stool | 7 | Urine leakage | 67 | Heat hypersensitivity in soles |
| 8 | Difficulty in excreting stools | 8 | Feeling of residual urine | 68 | Facial oedema |
| 9 | Taking laxatives | 9 | Enuresis | 69 | Oedema in hands |
| 10 | White / Yellow nasal discharge | 10 | Diarrhoea | 70 | Oedema in legs |
| 11 | Sticky nasal discharge | 11 | Haemorrhoid | 71 | Headache |
| 12 | Watery nasal discharge | 12 | Anal prolapse | 72 | Sluggishness |
| 13 | White / Yellow sputum | 13 | Bloody stool | 73 | Vertigo |
| 14 | Sticky sputum | 14 | Depressed mood | 74 | Light headedness |
| 15 | Watery sputum | 15 | Forgetfulness | 75 | Dandruff |
| 16 | Abdominal pain while fasting | 16 | Irritated | 76 | Hair loss |
| 17 | Abdominal pain after eating | 17 | Feel low | 77 | Decreased visual acuity |
| 18 | Upper abdominal pain | 18 | Short attention span | 78 | Eyestrain |
| 19 | Lower abdominal pain | 19 | Impatience | 79 | Blurred vision |
| 20 | Sudden abdominal pain | 20 | Hard to wake up | 80 | Bleary eyes |
| 21 | Heavy / Less menstrual flow | 21 | Easily surprised | 81 | Dark circles around the eyes |
| 22 | Irregular menstruation | 22 | Dry skin | 82 | Sneezing |
| 23 | Pill use | 23 | Itchy skin | 83 | Post nasal drip |
| 24 | Delivery | 24 | Acne | 84 | Stuffy nose |
| 25 | Spontaneous abortion | 25 | Blot | 85 | Nosebleed |
| 26 | Induced abortion | 26 | Urticaria | 86 | Bitter taste in mouth |
| 27 | Pregnancy toxaemia | 27 | Wart | 87 | Too much saliva comes out |
| 28 | Abnormal bleeding | 28 | Athlete's foot | 88 | Throat pain |
| 29 | Uterine prolapse | 29 | Brittle nails | 89 | Throat jamming comfort |
|  |  | 30 | Bruising | 90 | Thirsty |
|  |  | 31 | Easily fatigued | 91 | Dry mouth |
|  |  | 32 | Easy to sweat | 92 | Dry lips |
|  |  | 33 | Night sweats | 93 | Drink water often |
|  |  | 34 | Hot flashes | 94 | Tinnitus |
|  |  | 35 | Heat intolerance | 95 | Hearing loss |
|  |  | 36 | Cold intolerance | 96 | Cough |
|  |  | 37 | Attenuation of sexual desire | 97 | Asthma |
|  |  | 38 | Impotence | 98 | Shortness of breath |
|  |  | 39 | Easy to catch a cold | 99 | Palpitations |
|  |  | 40 | Feeling sluggish | 100 | Chest pain |
|  |  | 41 | Feeling of heaviness | 101 | Chest jamming discomfort |
|  |  | 42 | Change in symptoms in one day | 102 | Burping |
|  |  | 43 | Neck stiffness | 103 | Heartburn |
|  |  | 44 | Shoulder stiffness | 104 | Epigastric jamming discomfort |
|  |  | 45 | Back stiffness | 105 | Nausea |
|  |  | 46 | Lower back stiffness | 106 | Vomiting |
|  |  | 47 | Facial pain | 107 | Motion sickness |
|  |  | 48 | Hand pain | 108 | Stomach fullness |
|  |  | 49 | Foot pain | 109 | Stomach rumbling |
|  |  | 50 | Shoulder pain | 110 | Flatulence |
|  |  | 51 | Back pain | 111 | Sleepy after eating |
|  |  | 52 | Hip pain | 112 | Abdominal pain |
|  |  | 53 | Knee pain | 113 | Hand stiffness |
|  |  | 54 | Facial numbness | 114 | Lower extremity weakness |
|  |  | 55 | Hand numbness | 115 | Leg instability |
|  |  | 56 | Leg numbness | 116 | Leg spasms |
|  |  | 57 | Back numbness | 117 | Frost bite |
|  |  | 58 | Trembling face | 118 | Menstruation textile |
|  |  | 59 | Trembling hands | 119 | Menstrual pain |
|  |  | 60 | Trembling legs |  |  |
